# Factors associated with incomplete adherence to integrase strand transfer inhibitor-containing single-tablet regimen among Japanese people living with HIV

**DOI:** 10.1186/s40780-024-00349-7

**Published:** 2024-06-05

**Authors:** Yusuke Kunimoto, Shinichi Hikasa, Masashi Ishihara, Mariko Tsukiji, Kazuko Nobori, Takeshi Kimura, Kenta Onishi, Yuuki Yamamoto, Kyohei Haruta, Yohei Kasiwabara, Kenji Fujii, Masahide Fukudo

**Affiliations:** 1 Department of Pharmacy, Sapporo Medical University Hospital, Sapporo, Japan; 2 Department of Pharmacy, Hyogo Medical University Hospital, Nishinomiya, Japan; 3 Department of Pharmacy, Gifu University Hospital, Gifu, Japan; 4 Division of Pharmacy, Chiba University Hospital, Chiba, Japan; 5 Department of Pharmacy, Kobe City Medical Center General Hospital, Kobe, Japan; 6 Department of Pharmacy, Kobe University Hospital, Kobe, Japan; 7 Division of Pharmacy, Wakayama Medical University Hospital, Wakayama, Japan; 8 Division of Pharmacy, Himeji Medical Center, Himeji, Japan; 9 Department of Pharmacy, Nara Medical University Hospital, Kashihara, Japan; 10 Division of Pharmacy, Japanese Red Cross Kyoto Daiichi Hospital, Higashiyama, Japan; 11 Department of Pharmaceutical Services, Hiroshima University Hospital, Hiroshima, Japan

**Keywords:** HIV, Antiretroviral therapy, Antiretroviral drug, Integrase strand transfer inhibitor, Single-tablet regimen, ART adherence, Japan

## Abstract

**Background:**

People living with human immunodeficiency virus (PLWH) require high rates of medication adherence to antiretroviral therapy (ART) for a successful treatment outcome. Understanding the factors associated with incomplete adherence among those receiving integrase strand transfer inhibitor-containing single-tablet regimens (INSTI-STRs) is crucial for improving treatment outcomes. This study aimed to identify the factors contributing to incomplete ART adherence among Japanese PLWH receiving INSTI-STRs.

**Methods:**

This multicenter cross-sectional study was conducted at 11 Japanese institutions as an anonymous survey. ART adherence was assessed using a self-reported questionnaire. We defined incomplete ART adherence as missing ≥ 1 dose of antiretroviral drugs (ARVs) over the past month. The factors associated with incomplete ART adherence were assessed using logistic regression analysis. Additionally, we investigated the associations between patients’ satisfaction score with and need for ARVs and their adherence to ART.

**Results:**

The final analysis included data of 387 patients who were treated with INSTI-STRs. Multivariate logistic regression demonstrated significant association of younger age (adjusted odds ratio [aOR], 0.79; 95%confidence interval [CI]: 0.64–0.99 for each 10-year increment) with incomplete ART adherence. Additionally, female sex (aOR, 3.98; 95%CI: 1.36–11.60); depressive symptoms (mild depression: aOR, 1.68; 95%CI: 1.001–2.82, moderate depression: aOR, 2.98; 95%CI: 1.35–6.53, and severe depression: aOR, 8.73; 95%CI: 1.38–55.00 vs. minimal depression); were also significantly associated with incomplete ART adherence when compared with the reference categories. Concomitant medication usage was significantly associated with a lower rate of incomplete ART adherence (1–4 medications: aOR, 0.53; 95%CI: 0.31–0.89 and ≥ 5 medications: aOR, 0.30; 95%CI: 0.13–0.70 vs. no concomitant medication usage). In the incomplete ART adherence group, satisfaction scores for various aspects were significantly lower. Furthermore, a lower proportion of patients in the incomplete ART adherence group preferred the option of “taking tablets daily and visiting the hospital every 3 months,” compared to those in the complete ART adherence group (*p* = 0.008).

**Conclusions:**

This study demonstrated that factors associated with incomplete ART adherence include younger age, female sex, no concomitant medication, and depressive symptoms. Despite ART simplification, incomplete adherence among PLWH receiving INSTI-STRs, remains a challenge, requiring additional actions.

**Supplementary Information:**

The online version contains supplementary material available at 10.1186/s40780-024-00349-7.

## Background

People living with human immunodeficiency virus (PLWH) require extremely high levels of medication adherence to antiretroviral therapy (ART) for a successful treatment outcome [1]. In addition, several reports have indicated that ART non-adherence is associated with adverse clinical outcomes, such as residual inflammatory responses in PLWH, even when the HIV viral load is suppressed [2–4]. The simplification of ART since the advent of the single-tablet regimen (STR), which is a form of HIV treatment that combines multiple drugs into one daily pill, has positively impacted medication adherence and treatment satisfaction among PLWH [5–10]. According to current HIV treatment guidelines, integrase strand transfer inhibitor-containing STRs (INSTI-STRs) are primarily recommended as the initial treatment regimen [11]. Although some treatments are exceptions, most ARTs still require daily oral medication intake, and maintaining medication adherence is a critical therapeutic concern. Previous studies have indicated that incomplete ART adherence (< 100%) can negatively impact patients’ prognosis and inflammatory status [2, 12]. Therefore, aiming for complete ART adherence may provide greater benefits to PLWH.

ART non-adherence has been reported to be associated with age, sex, education level, employment status, treatment factors, disease factors, patients’ satisfaction with ART, and mental health status [6, 13–19]. However, no studies have focused on INSTI-STRs, which are currently the mainstream treatment strategies. Furthermore, each country or region may have characteristics that may serve as barriers to treatment, and limited studies exist on the factors contributing to ART non-adherence in Japanese patients [13]. Consequently, it is essential to identify individual regional factors associated with ART non-adherence. This study aimed to investigate the factors associated with incomplete ART adherence among Japanese PLWH, receiving treatment with INSTI-STRs. Additionally, we investigated the relationships between patients’ satisfaction with antiretroviral drugs (ARVs), their needs for ARVs, and ART adherence.

## Methods

### Study design and patients

This multicenter cross-sectional study was an anonymous survey conducted from April 2021 to December 2021 through self-reported questionnaires distributed at 11 institutions across Japan. Patients eligible for this study had to meet all of the following criteria: 1) aged 20 years or older, 2) having reading and writing ability in Japanese, and 3) have been receiving current ART for more than 4 weeks. Patients with unanswered questions, those with erroneous information, or those who did not provide consent to participate in this study were excluded. All study participants provided written informed consent. Questionnaires were distributed manually. Participation in the survey was voluntary, and confidentiality was maintained throughout the study and analysis. The protocol of this study was approved by the institutional review board at Sapporo Medical University (322–301) and all other participating research centers.

### Measurements

#### Medication adherence to ART

Medication adherence to ART was assessed using self-reported questions on the frequency of missing ARV doses over the past month. Complete ART adherence was defined as having no missing doses of ARVs, while incomplete ART adherence was defined as having missed one or more doses of ARVs.

#### HIV Treatment Satisfaction Questionnaire: Status (HIVTSQs)

Data were collected in patient-completed questionnaire forms using the Japanese versions of the HIVTSQs for assessing satisfaction with ART [20–22]. Each item on the HIVTSQs was scored on a Likert scale ranging from 0 (least satisfied) to 6 (most satisfied). We calculated the total score for the HIVTSQs by summing the scores for items 1–11, resulting in a possible range of 0–66. The higher and lower scores indicated more and less satisfaction with treatment, respectively.

#### Patient Health Questionnaire-9 (PHQ-9)

Depressive symptoms were measured using the Japanese versions of the PHQ-9 [23–25]. The PHQ-9 is a nine-item questionnaire assessing depressive symptoms over the past 2 weeks, with participants rating each symptom’s frequency on a scale from 0 (not at all) to 3 (nearly every day). The scores for the PHQ-9 were calculated as the sum of the score for each item, with a total score ranging from 0 to 27. The total scores on the PHQ-9 were classified according to levels of depression severity as follows: 0–4 (minimal), 5–9 (mild), 10–14 (moderate), 15–19 (moderately severe), and 20–27 (severe). Higher scores indicated greater depressive symptoms.

#### Satisfaction with the ARV questionnaire

The questionnaire, designed specifically for assessing satisfaction with ARV use includes seven items covering different aspects, such as size, feeling while receiving ARV, color, taste, portability, daily oral therapy, and co-payment [26]. Patients responded to the question of “How satisfied are you with the anti-HIV medications you are currently taking?” on a 7-point Likert scale (0 = not satisfied at all, 1 = not satisfied, 2 = somewhat unsatisfied, 3 = good, 4 = somewhat satisfied, 5 = satisfied, and 6 = very satisfied), to indicate their satisfaction with using ARVs.

#### Questionnaire on the needs for future drug formulation for PLWH taking ARVs

PLWH needs were assessed using a tailored questionnaire focusing on future ARV formulation, considering factors such as dosage form and dosing frequency [26]. Patients responded to the question, “What type of anti-HIV drugs would you like to use based on your experience?” by selecting from a list of options that reflected their treatment preferences.

### Data collection

Data collection was conducted on the date of questionnaire completion, with all information sourced from the patients' hospital's electronic medical records. Self-administered questionnaires were completed by patients and submitted or collected via mail. Demographic and clinical data included age, sex, education level, employment status, CD4 cell count, HIV RNA levels, history of acquired immunodeficiency syndrome diagnosis, time on ART, current ART regimen, and use of concomitant medications. These variables were selected based on previous investigations on factors associated with ART adherence [14, 16, 17, 19]. Education level was divided into two categories: vocational school/college of technology/university and lower; HIV RNA levels were classified into < 50 and ≥ 50 copies/mL groups; current ART regimen was categorized based on the type of INSTI and the presence of dietary restrictions; and the dietary requirements of the regimen were classified as food required and not required with ART. Duration of ART was classified as, < 5, 5–9, and ≥ 10 years), and the number of concomitant medications as 0, 1–4, and ≥ 5, both based on categories used in previous studies [27, 28].

### Statistical methods

Continuous data are presented as medians (interquartile ranges). Categorical variables are presented as frequencies and percentages. The Mann–Whitney *U*-test was used to compare continuous variables. The chi-square test or Fisher’s exact test was used to compare categorical variables. Binary logistic regression analysis was performed to examine associations between demographics, clinical data, HIVTSQs score, PHQ-9 score, and incomplete ART adherence. Subsequently, factors exhibiting a potentially significant level (*p* < 0.1) in the binomial logistic regression analysis were included in the multivariate model. Notably, the variance inflation factors (VIFs) of all variables to be included in the multivariate logistic regression analysis were evaluated, and all variables with VIF > 5 were excluded. Complete case analysis was performed because the missing data were < 5% [29]. Statistical significance was defined as *p* < 0.05 for a two-sided test. All statistical analyses were performed using JMP Pro version 15 (SAS Institute, Cary, NC, USA).

## Results

### Demographic and clinical characteristics

Among the 704 patients who agreed to participate in this study, data from 387 patients using INSTI-STRs with no unanswered or erroneous responses to the adherence question were included in the final analysis. A flowchart of the enrollment of study patients is presented in Supplemental Figure S1 in Additional file 1. Table 1 shows the demographic and clinical characteristics of the study patients. The treatment regimen for 387 patients is detailed in Supplemental Table S1 in Additional file 2. The mean age of the patients was 47.5 years. Most patients were male (95.6%), and HIV-RNA was undetectable (< 50 copies/mL) in most individuals (97.4%).

**Table 1:** Demographic and clinical characteristics^a^

Characteristics	
Number of patients	387
Age (years), median (IQR)	47.0 (39.0–54.0)
Sex, n (%)	
Male	370 (95.6)
Female	17 (4.4)
Education level^b^, n (%)	
Primary school/Secondary school	146 (37.7)
Vocational school/College of Technology/University	239 (61.8)
Missing data	2 (0.5)
Employment status^b^, n (%)	
Employed	308 (79.6)
Unemployed	63 (16.3)
Missing data	16 (4.1)
CD4 cell count (cells/µL), median (IQR)	570 (434–752)
HIV-RNA < 50 copies/mL, n (%)	
Yes	377 (97.4)
No	10 (2.6)
Prior AIDS diagnosis^b^, n (%)	
Yes	124 (32.0)
No	262 (67.7)
Missing data	1 (0.3)
Time on ART (years), n (%)	
< 5	121 (31.3)
5–9	122 (31.5)
≥ 10	144 (37.2)
INSTIs in the current regimen^c^, n (%)	
Bictegravir	203 (52.5)
Dolutegravir	162 (41.9)
Elvitegravir	22 (5.7)
Food requirements for the regimen, n (%)	
Yes	30 (7.8)
No	357 (92.2)
Number of concomitant medications, n (%)	
None	187 (48.3)
1–4	143 (37.0)
≥ 5	57 (14.7)
Total score of HIVTSQs^b^, median (IQR)	59 (53–65)
Depression level^d^, n (%)	
Minimal	197 (50.9)
Mild	127 (32.8)
Moderate	36 (9.3)
Moderately severe	20 (5.2)
Severe	7 (1.8)

*Abbreviations*: *AIDS* Acquired immunodeficiency syndrome, *ART* Antiretroviral therapy, *INSTI* Integrase strand transfer inhibitor, *HIVTSQs* HIV treatment satisfaction questionnaire: status, *PHQ-9* Patient health questionnaire-9

^a^Data are expressed as number (%) or median (IQR)

^b^Missing data/nonresponses were excluded for the following variables: educational level (*n* = 2), employment status (*n* = 16), prior AIDS diagnosis (*n* = 1), and total score of HIVTSQs (*n* = 6)

^c^The sum may not equal 100% due to rounding to the second decimal place for each item in the table

^d^The total scores on the PHQ-9 were classified according to levels of depression severity as follows: 0–4 (minimal), 5–9 (mild), 10–14 (moderate), 15–19 (moderately severe), and 20–27 (severe)

### Binary logistic regression analysis of factors associated with incomplete ART adherence

Incomplete ART adherence was observed in 34.1% of patients. The results of univariate and multivariate logistic regression analyses are presented in Table 2. Univariate analysis demonstrated significant association of younger age (odds ratio [OR] 0.68, 95%confidence interval [CI]: 0.56–0.82 for each 10-year increment, *p* < 0.001), female sex (OR 2.90, 95%CI: 1.08–7.81, *p* = 0.035), higher CD4 cell count (OR 1.11, 95%CI: 1.02–1.21 for each 100 cells/µL increment, *p* = 0.014), lower total HIVTSQs score (OR 0.97, 95%CI: 0.94–0.99 for each 1-point increment, *p* = 0.011), depressive symptoms (moderate depression: OR 2.37, 95%CI: 1.15–4.89, *p* = 0.020 and severe depression: OR 6.62, 95%CI: 1.25–35.15, *p* = 0.027 vs minimal depression) with incomplete ART adherence. Concomitant medication was significantly associated with a lower rate of incomplete ART adherence (using 1–4: OR 0.46, 95%CI: 0.29–0.74, *p* = 0.001 and using ≥ 5: OR 0.34, 95%CI: 0.17–0.69, *p* = 0.003).

**Table 2:** Univariate and multivariate logistic regression analysis of the factors associated with incomplete ART adherence

Variable	Complete ART adherence (*n* = 255)	Incomplete ART adherence (*n* = 132)	Unadjusted OR (95% CI)	*p* value^a^	Adjusted OR (95% CI)	*p* value^a^
Age (years)^b^, median (IQR)	48.0 (41.0–56.0)	44.5 (36.0–49.0)	0.68 (0.56–0.82)	< 0.001	0.79 (0.64–0.99)	0.038
Sex, n (%)				0.035		0.012
Male	248 (97.3)	122 (92.4)	1.00 (reference)		1.00 (reference)	
Female	7 (2.7)	10 (7.6)	2.90 (1.08–7.81)		3.98 (1.36–11.60)	
Education level ^c^, n (%)				0.649		
Primary school/Secondary school	98 (38.4)	48 (36.4)	0.90 (0.58–1.40)			
Vocational school/College of Technology/University	155 (60.8)	84 (63.6)	1.00 (reference)			
Missing data	2 (0.8)	0				
Employment status ^c,d^, n (%)				0.300		
Employed	199 (78.0)	109 (82.6)	1.00 (reference)			
Unemployed	45 (17.6)	18 (13.6)	0.73 (0.40–1.32)			
Missing data	11 (4.3)	5 (3.8)				
CD4 cell count (cells/µL) ^b^, median (IQR)	565 (404–732)	616.5 (477.5–786.5)	1.11 (1.02–1.21)	0.014	1.09 (0.997–1.20)	0.058
HIV-RNA < 50 copies/mL, n (%)				0.691		
Yes	249 (97.6)	128 (97.0)	1.00 (reference)			
No	6 (2.4)	4 (3.0)	1.30 (0.36–4.68)			
Prior AIDS diagnosis ^c^, n (%)				0.104		
Yes	89 (34.9)	35 (26.5)	1.00 (reference)			
No	166 (65.1)	96 (72.7)	1.47 (0.92–2.34)			
Missing data	0	1 (0.8)				
Time on ART (years), n (%)				0.612		
< 5	76 (29.8)	45 (34.1)	1.15 (0.69–1.90)	0.592		
5–9	84 (32.9)	38 (28.8)	0.88 (0.52–1.47)	0.618		
≥ 10	95 (37.3)	49 (37.1)	1.00 (reference)			
INSTIs in the current regimen, n (%)				0.425		
Bictegravir	138 (54.1)	65 (49.2)	1.00 (reference)			
Dolutegravir	105 (41.2)	57 (43.2)	1.15 (0.74–1.78)	0.524		
Elvitegravir	12 (4.7)	10 (7.6)	1.77 (0.73–4.31)	0.209		
Food requirements for the regimen, n (%)				0.758		
Yes	19 (7.5)	11 (8.3)	1.13 (0.52–2.45)			
No	236 (92.5)	121 (91.7)	1.00 (reference)			
Number of concomitant medications, n (%)				< 0.001		0.006
None	105 (41.2)	82 (62.1)	1.00 (reference)		1.00 (reference)	
1–4	105 (41.2)	38 (28.8)	0.46 (0.29–0.74)	0.001	0.53 (0.31–0.89)	0.016
≥ 5	45 (17.6)	12 (9.1)	0.34 (0.17–0.69)	0.003	0.30 (0.13–0.70)	0.005
Total score of HIVTSQs ^b,c^, median (IQR)	60 (54–65)	58 (51–63)	0.97 (0.94–0.99)	0.011	0.98 (0.95–1.01)	0.147
Depression level ^d,e^, n (%)				0.020		0.010
Minimal	143 (56.1)	54 (40.9)	1.00 (reference)		1.00 (reference)	
Mild	80 (31.4)	47 (35.6)	1.56 (0.97–2.51)	0.070	1.68 (1.001–2.82)	0.0496
Moderate	19 (7.5)	17 (12.9)	2.37 (1.15–4.89)	0.020	2.98 (1.35–6.53)	0.007
Moderately severe	11 (4.3)	9 (6.8)	2.17 (0.85–5.52)	0.105	2.70 (0.97–7.52)	0.058
Severe	2 (0.8)	5 (3.8)	6.62 (1.25–35.15)	0.027	8.73 (1.38–55.00)	0.021

*Abbreviations*: *AIDS* Acquired immunodeficiency syndrome, *ART* Antiretroviral therapy, *CI* Confidence interval, *INSTI* Integrase strand transfer inhibitor, *HIVTSQs* HIV treatment satisfaction questionnaire: status, *PHQ-9* Patient health questionnaire-9, *OR* Odds ratio

^a^Wald test

^b^Odds ratio are expressed as follows: age per 10-year increment, CD4 cell count per 100 cells/µL increment, total score of HIVTSQs per 1-point increment

^c^Missing data/nonresponses were excluded for the following variables: educational level (*n* = 2), employment status (*n* = 16), prior AIDS diagnosis (*n* = 1), and total score of HIVTSQs (*n* = 6)

^d^The sum may not equal 100% due to rounding to the second decimal place for each item in the table

^e^The total scores on the PHQ-9 were classified according to levels of depression severity as follows: 0–4 (minimal), 5–9 (mild), 10–14 (moderate), 15–19 (moderately severe), and 20–27 (severe)

Multivariate logistic regression analysis was performed by including age, sex, CD4 cell count, number of concomitant medications, total score of HIVTSQs, and level of depression as confounding variables. The mean VIF value for variables in the multivariate analysis was 1.17 (range: 1.02–1.51), indicating a reasonable level of multicollinearity. The multivariate logistic regression revealed the following factors to be significantly associated with incomplete ART adherence; younger age (adjusted OR [aOR] 0.79, 95%CI: 0.64–0.99 for each 10-year increment, *p* = 0.038), female sex (aOR 3.98, 95%CI: 1.36–11.60, *p* = 0.012), mild depression (aOR 1.68, 95%CI: 1.001–2.82, *p* = 0.0496), moderate depression (aOR 2.98, 95%CI: 1.35–6.53, *p* = 0.007), and severe depression (aOR 8.73, 95%CI: 1.38–55.00, *p* = 0.021). Concomitant medication was associated with a lower rate of incomplete ART adherence (using 1–4: aOR 0.53, 95%CI: 0.31–0.89, *p* = 0.016 and using ≥ 5: aOR 0.30, 95%CI: 0.13–0.70, *p* = 0.005).

### Comparison of ARV satisfaction and future preference for ARV of PLWH between complete and incomplete ART adherence groups

**Fig. 1:**
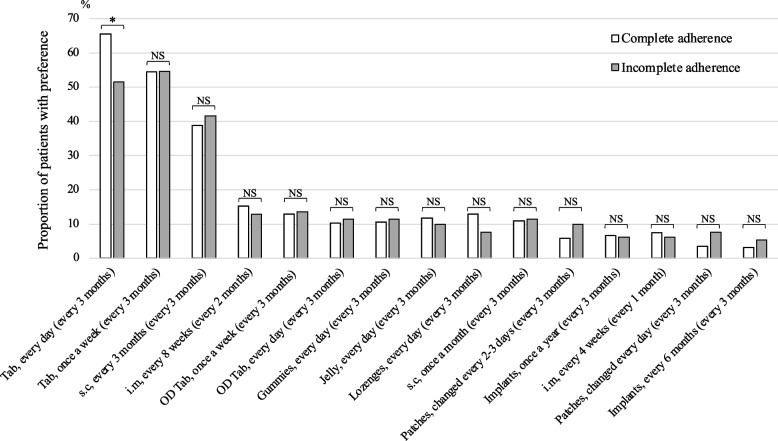
Comparison of PLWH with complete and incomplete ART adherence regarding their future ARV preferences (*n* = 387). The questionnaire allowed respondents to choose their preferred ARVs, with multiple responses possible. Each item indicates the formulation of a drug, frequency of use, (frequency of hospital visit). Asterisks indicate statistical significance between the two groups, as determined by the chi-square test (NS, nonsignificant; *, *p* < 0.01). ARVs, anti-retroviral drugs; Tab, tablets; s.c, subcutaneous injection; i.m, intramuscular injection; OD, orally disintegrating tablets; PLWH, people living with HIV; ART, anti-retroviral therapy

The results of the comparison between PLWH in the complete and incomplete ART adherence groups using a questionnaire on satisfaction with ARVs are shown in Table 3. In the incomplete ART adherence group, ARV satisfaction scores for size (*p* = 0.043), feeling while receiving ARV (*p* = 0.002), taste (*p* = 0.012), portability (*p* = 0.004), and daily oral therapy (*p* < 0.001) were significantly lower than those of patients in the complete ART adherence group. The results of the comparison between PLWH in the two groups using a questionnaire regarding their future preference for ARVs are shown in Fig. 1 and Supplemental Table S2 in Additional file 2. In the incomplete ART adherence group, the proportion of patients who preferred “taking tablets daily and visiting the hospital every 3 months” was significantly lower (complete ART adherence: 65.5% and incomplete ART adherence: 51.5%; *p* = 0.008).

**Table 3:** Comparisons of satisfaction score of the current ART between the complete and incomplete ART adherence groups

Item label^a^	ART adherence	Satisfaction score^b^ / Number (%)^c^							Median (IQR)	*p* value^d^
		0	1	2	3	4	5	6		
Size	Complete (*n* = 252)	4 (1.6)	2 (0.8)	9 (3.6)	25 (9.9)	45 (17.9)	60 (23.8)	107 (42.5)	5 (4–6)	0.043
	Incomplete (*n* = 131)	5 (3.8)	1 (0.8)	6 (4.6)	21 (16.0)	27 (20.6)	23 (17.6)	48 (36.6)	5 (3–6)	
Feeling while using	Complete (*n* = 252)	2 (0.8)	2 (0.8)	6 (2.4)	18 (7.1)	45 (17.9)	70 (27.8)	109 (43.3)	5 (4–6)	0.002
	Incomplete (*n* = 131)	0 (0)	5 (3.8)	6 (4.6)	19 (14.5)	26 (19.8)	34 (26.0)	41 (31.3)	5 (4–6)	
Color	Complete (*n* = 251)	6 (2.4)	2 (0.8)	3 (1.2)	25 (10.0)	27 (10.8)	59 (23.5)	129 (51.4)	6 (4–6)	0.052
	Incomplete (*n* = 130)	3 (2.3)	1 (0.8)	3 (2.3)	20 (15.4)	13 (10.0)	37 (28.5)	53 (40.8)	5 (4–6)	
Taste	Complete (*n* = 251)	1 (0.4)	0 (0)	1 (0.4)	28 (11.2)	21 (8.4)	55 (21.9)	145 (57.8)	6 (5–6)	0.012
	Incomplete (*n* = 131)	2 (1.5)	1 (0.8)	6 (4.6)	17 (13.0)	14 (10.7)	30 (22.9)	61 (46.6)	5 (4–6)	
Portability	Complete (*n* = 252)	3 (1.2)	7 (2.8)	8 (3.2)	21 (8.3)	40 (15.9)	55 (21.8)	118 (46.8)	5 (4–6)	0.004
	Incomplete (*n* = 131)	4 (3.1)	6 (4.6)	8 (6.1)	15 (11.5)	24 (18.3)	30 (22.9)	44 (33.6)	5 (3–6)	
Daily oral therapy	Complete (*n* = 253)	4 (1.6)	5 (2.0)	7 (2.8)	41 (16.2)	44 (17.4)	56 (22.1)	96 (37.9)	5 (4–6)	< 0.001
	Incomplete (*n* = 131)	7 (5.3)	8 (6.1)	15 (11.5)	21 (16.0)	30 (22.9)	27 (20.6)	23 (17.6)	4 (3–5)	
Co-payment	Complete (*n* = 253)	1 (0.4)	3 (1.2)	6 (2.4)	29 (11.5)	22 (8.7)	52 (20.6)	140 (55.3)	6 (5–6)	0.810
	Incomplete (*n* = 131)	1 (0.8)	3 (2.3)	8 (6.1)	15 (11.5)	9 (6.9)	20 (15.3)	75 (57.3)	6 (4–6)	

*ART* Anti-retroviral therapy

^a^Missing data/nonresponses were excluded for the following items: size (*n* = 4), feeling while using (*n* = 4), color (*n* = 6), taste (*n* = 5), portability (*n* = 4), daily oral therapy (*n* = 3), and co-payment (*n* = 3)

^b^Each item is rated on a Likert scale ranging from 0 (very dissatisfied) to 6 (very satisfied), with higher scores indicating greater satisfaction

^c^The sum may not equal 100% due to rounding to the second decimal place for each item in the table

^d^Mann–Whitney U test

## Discussion

In this multicenter cross-sectional study spanning 11 institutions, we identified the factors associated with incomplete ART adherence among Japanese PLWH using INSTI-STRs. These include younger age, female sex, no concomitant medications, and depressive symptoms. Furthermore, patients with incomplete ART adherence were significantly less likely to prefer daily oral ART in pill form and expressed lower satisfaction with various current ARVs than those with complete ART adherence.

The results of the present study are similar to those of previous reports on PLWH receiving ART that are not limited to INSTI-STRs [6, 15, 30]. These findings suggest a consistent influence of the identified factors on ART non-adherence, irrespective of the treatment regimen or study region. Additionally, the use of concomitant medications was associated with complete ART adherence, which is consistent with the previous findings by a multicenter randomized trial [14]. Other reports have indicated inconsistent associations between polypharmacy and ART non-adherence [31, 32]. Our results showed that patients with polypharmacy (number of concomitant medications ≥ 5) were less likely to have incomplete ART adherence. Differences in the association of ART adherence between studies may be attributed to variations in adherence measurement methods and definitions, as well as differences in the type of ART regimen. Our results suggest that a more cautious approach to treatment contributes to preventing missed doses in patients with comorbidities as compared to those taking ARVs alone [33]. In our study, 34.1% of PLWH reported missed doses in the past month, even when treated with a simplified regimen, such as INSTI-STRs. In two previous studies, self-reported incomplete ART adherence rates in the past month were reported as 17% and 24%, respectively [34, 35]. Thus, ART adherence in our study is considered relatively low. There is potential for improved outcomes of achieving complete adherence [2], and our results highlight the need for additional interventions to maintain adherence.

The proportion of patients in the incomplete ART adherence group who preferred “taking tablets daily and visiting the hospital every 3 months” was significantly lower than that of those in the complete ART adherence group. Furthermore, despite a ceiling effect that should be interpreted with caution, satisfaction scores with the size, feeling while using ARVs, taste, portability, and daily oral dosing of ARVs were significantly lower in the incomplete ART adherence group than in the complete ART adherence group. Despite the simplicity and convenience of using INSTI-STRs, our findings indicate that some patients may not be optimally treated, highlighting the need for refinement in treatment strategies. A previous study reported that the burden regarding medication was associated with poor medication adherence [18]. Our results emphasize the importance of identifying patients’ needs and dissatisfaction with ARVs, as well as their ART adherence, in routine practice. For some patients dissatisfied with oral ART, switching to parenteral ART may have a positive impact [36, 37]. Similarly, switching from one STR to another may also reduce dissatisfaction, considering the various tablet forms used.

The factors identified for incomplete ART adherence may have a similar impact on patients using INSTI-STRs in regions other than Japan. However, our results cannot be extrapolated to regions where INSTI-STR is not widely used. Nevertheless, our results would provide important insights into ART adherence. Notably, we identified that younger age, female sex, no concomitant medications, and depressive symptoms influenced incomplete ART adherence among PLWH receiving treatment with INSTI-STRs. Our results suggest that these factors should be considered in addressing ART non-adherence in introducing INSTI-STRs. Furthermore, the present results emphasize the importance of considering the individual characteristics and preferences of patients when choosing ART. Our findings may serve as foundational data for improved adherence to INSTI-STR treatment in regions where its use is not widespread.

This study had some limitations. First, medication adherence was assessed with only a self-reported questionnaire, which may have led to a potential overestimation of adherence [38]. However, self-reported medication adherence measures have been widely used in previous clinical trials and have demonstrated associations with clinical outcomes in PLWH [39, 40]. Second, this study’s cross-sectional design impedes the determination of long-term trends in medication adherence, necessitating a longitudinal study for further clarification. Third, there exists the possibility of missing data that are not recorded in the hospital database.

Nonetheless, this study identified several factors associated with incomplete ART adherence among PLWH using INSTI-STRs. Additionally, dissatisfaction with ARVs may affect ART adherence in this population. Medical providers should pay attention to ART adherence when following up with patients exhibiting characteristics associated with non-adherence to INSTI-STRs. Routine assessment of patient needs and level of satisfaction with ARVs, along with the evaluation of ART adherence, may assist in assessing the appropriateness of continuing current therapy.

## Conclusions

The present study clarified that adherence to ART among Japanese PLWH treated with INSTI-STRs was still incomplete despite the simplification of ART, which remains a challenge. We identified factors associated with incomplete ART adherence among Japanese PLWH receiving treatment with INSTI-STRs, including younger age, female sex, no concomitant medications, and symptoms of depression.

### Supplementary Information

Additional file 1: Supplemental Figure S1. Patient enrollment flowchart.

Additional file 2: Supplemental Table S1. Antiretroviral therapy regimens included in the study. Supplemental Table S2. Comparison of PLWH with complete and incomplete ART adherence and their future ARV preferences.

## Data Availability

All data generated or analyzed during this study are included in this published article and its supplementary information files.

## Abbreviations

PLWH: People living with HIV
ART: Antiretroviral therapy
INSTI-STR: Integrase strand transfer inhibitor-containing single-tablet regimen
HIV: Human immunodeficiency virus
ARV: Antiretroviral drug
STR: Single-tablet regimen
HIVTSQs: HIV treatment satisfaction questionnaire: status
PHQ-9: Patient health questionnaire-9
VIF: Variance inflation factor
OR: Odds ratio
aOR: Adjusted odds ratio
INSTI: Integrase strand transfer inhibitor
CI: Confidence interval
