# Supplementary material for: Factors associated with incomplete adherence to integrase strand transfer inhibitor-containing single-tablet regimen among Japanese people living with HIV
Source: J Pharm Health Care Sci. 2024 Jun 5;10:27. doi: 10.1186/s40780-024-00349-7 (PMC11151521; doi:10.1186/s40780-024-00349-7)
Supplement: Supplementary file 1 — Additional file 1: Supplemental Figure S1. Patient enrollment flowchart. [file 40780_2024_349_MOESM1_ESM.pptx]

## Slide 1
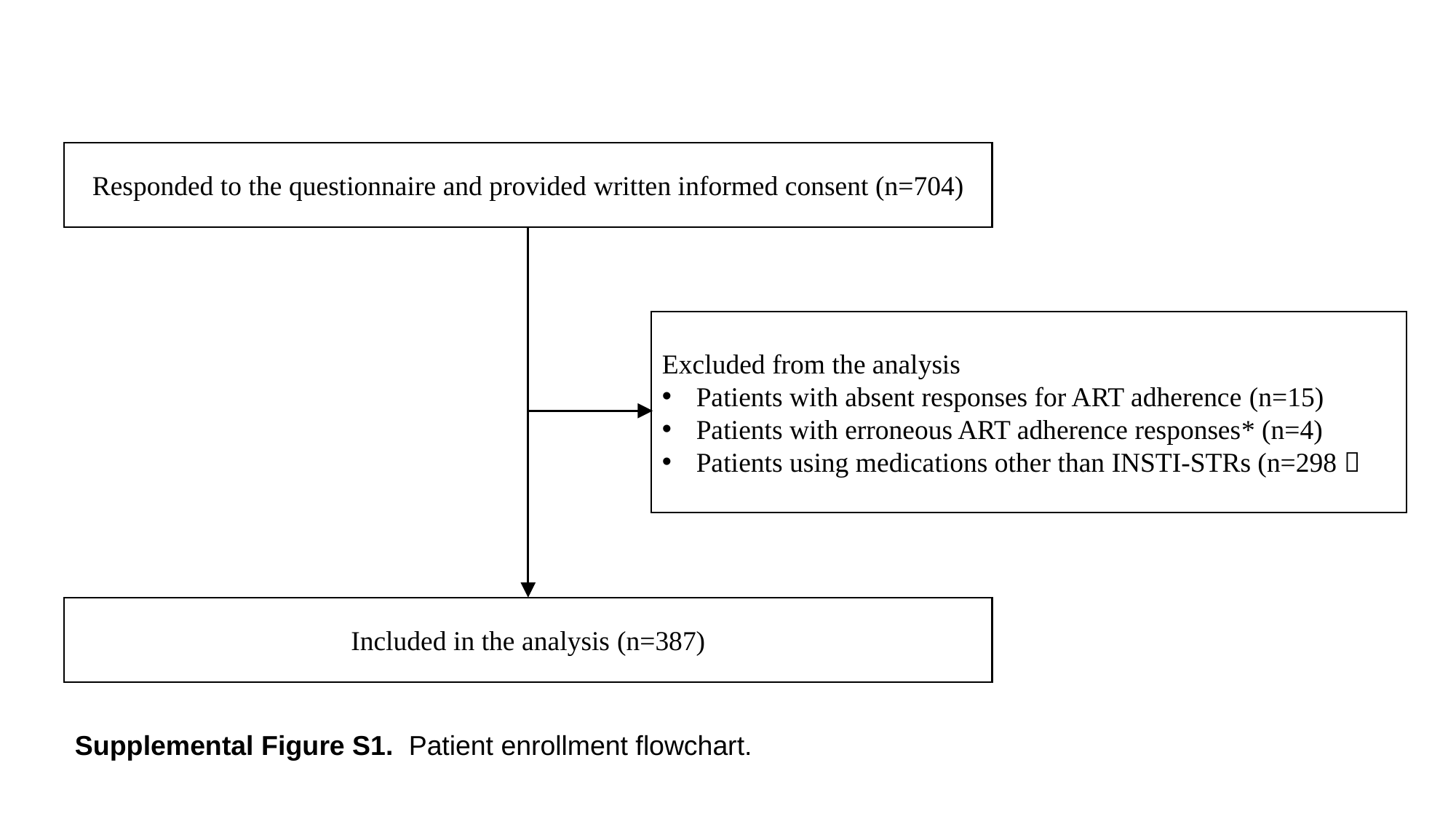

Responded to the questionnaire and provided written informed consent (n=704)
Excluded from the analysis
Patients with absent responses for ART adherence (n=15)
Patients with erroneous ART adherence responses* (n=4)
Patients using medications other than INSTI-STRs (n=298）
Included in the analysis (n=387)
Supplemental Figure S1. Patient enrollment flowchart.
