# Supplementary material for: Factors associated with incomplete adherence to integrase strand transfer inhibitor-containing single-tablet regimen among Japanese people living with HIV
Source: J Pharm Health Care Sci. 2024 Jun 5;10:27. doi: 10.1186/s40780-024-00349-7 (PMC11151521; doi:10.1186/s40780-024-00349-7)
Supplement: Supplementary file 2 — Additional file 2: Supplemental Table S1. Antiretroviral therapy regimens included in the study. Supplemental Table S2. Comparison of PLWH with complete and incomplete ART adherence and their future ARV preferences. [file 40780_2024_349_MOESM2_ESM.docx]

**Supplemental Table S1.** Antiretroviral therapy regimens included in the study.

| Regimen | Number (n = 387) | Percentage (%) ^a^ |
| --- | --- | --- |
| BIC/TAF/FTC | 203 | 52.5 |
| DTG/ABC/3TC | 102 | 26.4 |
| DTG/3TC | 52 | 13.4 |
| EVG/COBI/TAF/FTC | 21 | 5.4 |
| DTG/RPV | 8 | 2.1 |
| EVG/COBI/TDF/FTC | 1 | 0.3 |

Abbreviations: ABC, abacavir; BIC, bictegravir; COBI, cobicistat; DTG, dolutegravir; FTC, emtricitabine; EVG, elvitegravir; RPV, rilpivirine; TAF, tenofovir alafenamide fumarate; TDF, tenofovir disoproxil fumarate; 3TC, lamivudine

^a^ The sum may not equal 100% due to rounding to the second decimal place for each item in the table.

**Supplemental Table S2.** Comparison of PLWH with complete and incomplete ART adherence and their future ARV preferences.

| Item label | Complete ART adherence (n = 255),  n (%) | Incomplete ART adherence (n = 132),  n (%) | *p* value ^a^ |
| --- | --- | --- | --- |
| Drug formulation, frequency of use (frequency of hospital visit) |  |  |  |
| Tab, every day (every 3 months) | 167 (65.5) | 68 (51.5) | 0.008 |
| Tab, once a week (every 3 months) | 139 (54.5) | 72 (54.5) | 0.995 |
| s.c, every 3 months (every 3 months) | 99 (38.8) | 55 (41.7) | 0.588 |
| i.m, every 8 weeks (every 2 months) | 39 (15.3) | 17 (12.9) | 0.522 |
| OD Tab, once a week (every 3 months) | 33 (12.9) | 18 (13.6) | 0.848 |
| OD Tab, every day (every 3 months) | 26 (10.2) | 15 (11.4) | 0.724 |
| Gummies, every day (every 3 months) | 27 (10.6) | 15 (11.4) | 0.816 |
| Jelly, every day (every 3 months) | 30 (11.8) | 13 (9.8) | 0.570 |
| Lozenges, every day (every 3 months) | 33 (12.9) | 10 (7.6) | 0.111 |
| s.c, once a month (every 3 months) | 28 (11.0) | 15 (11.4) | 0.910 |
| Patches, changed every 2-3 days (every 3 months) | 15 (5.9) | 13 (9.8) | 0.153 |
| Implants, once a year (every 3 months) | 17 (6.7) | 8 (6.1) | 0.818 |
| i.m, every 4 weeks (every 1 month) | 19 (7.5) | 8 (6.1) | 0.611 |
| Patches, changed every day (every 3 months) | 9 (3.5) | 10 (7.6) | 0.081 |
| Implants, every 6 months (every 3 months) | 8 (3.1) | 7 (5.3) | 0.295 |

Abbreviations: ARVs, anti-retroviral drugs; Tab, tablets; s.c, subcutaneous injection; i.m, intramuscular injection; OD, orally disintegrating tablets; PLWH, people living with HIV; ART, anti-retroviral therapy

^a^ Chi-square test
